# Tissue factor-associated neutrophil extracellular traps in dermatomyositis: procoagulant activity and endothelial activation *in vitro*

**DOI:** 10.3389/fimmu.2026.1926259

**Published:** 2026-10-30

**Authors:** Maria Ntinopoulou, Lamprini Efstathiou, Aliki I. Venetsanopoulou, Pavlos Simonopoulos, Evangeli Lampri, Nikolaos Koletsos, Nikolaos Zintziovas, Antigoni Sourla, Paraskevi V. Voulgari, Akrivi Chrysanthopoulou

**Affiliations:** 1 Laboratory of Molecular Immunology, Department of Biological Applications & Technology, Faculty of Health Sciences, University of Ioannina, Ioannina, Greece; 2 Department of Pathology, University Hospital of Ioannina, Ioannina, Greece; 3 Rheumatology Clinic, Department of Internal Medicine, Faculty of Medicine, School of Health Sciences, University of Ioannina, Ioannina, Greece; 4 Pathology Department, Bioiatriki Laboratories, Athens, Greece

**Keywords:** complement, dermatomyositis, inflammation, NETs, neutrophils, tissue factor

## Abstract

**Introduction:**

Dermatomyositis (DM) is an immune-mediated myopathy characterized by inflammatory and vascular manifestations and variable systemic involvement; however, the interplay among neutrophil activation, complement, and coagulation remains unclear. We investigated whether serum from patients with DM induces tissue factor (TF)-associated neutrophil extracellular traps (NETs) and examined their functional effects *in vitro*.

**Methods:**

Serum from seven patients with active DM and seven matched healthy individuals was analyzed for inflammatory mediators, citrullinated histone H3 (CitH3), thrombin–antithrombin (TAT) complexes, soluble C5b-9, and DNase-I activity. Control neutrophils were exposed to DM serum and, then, NET formation, TF expression and activity, and thrombin generation were assessed. Inhibition experiments targeting complement, TF, and NETs were performed. Co-culture experiments assessed endothelial activation, while skin biopsies were examined by immunofluorescence.

**Results:**

Patients with DM exhibited increased inflammatory mediators, CitH3, TAT complexes, and soluble C5b-9, together with reduced DNase-I activity. DM serum induced TF expression and release of TF-associated NETs by control neutrophils. DNase-I and eculizumab reduced NET formation, while eculizumab additionally decreased TF mRNA expression and NET-associated TF activity. TF-associated NETs promoted adhesion molecule expression by human aortic endothelial cells, an effect attenuated by DNase-I or TF neutralization. TF-positive NET fragments were detected in lesional DM skin but not healthy skin.

**Discussion:**

These exploratory findings support a contribution of C5-dependent complement activation to TF-associated NET formation and show that these NETs can enhance thrombin generation and endothelial activation *in vitro*. Their relationship to clinical vasculopathy or thrombotic risk remains unknown, providing evidence for potential links between inflammation, thrombosis, and vascular injury in DM.

## Introduction

Dermatomyositis (DM) is a heterogeneous immune-mediated disease characterized by cutaneous inflammation, with or without muscle involvement, and variable systemic manifestations, including interstitial lung disease (1–3). Vascular abnormalities are also part of its pathological spectrum. Capillary loss and perifascicular changes are observed in muscle, while endothelial alterations, periungual abnormalities, erythema, and, in more severe cases, cutaneous ulceration may occur in the skin (4, 5). However, the mechanisms linking inflammation to endothelial and coagulation-related responses in DM remain incompletely defined.

Type I interferon signaling and complement activation are two well-established components of DM pathobiology. Type I interferon–stimulated gene signatures are detected in affected tissues and peripheral blood and may contribute to persistent inflammation and vascular changes (6, 7). Type I interferon signaling is largely mediated through the Janus kinase–signal transducer and activator of transcription (JAK/STAT) pathway, and JAK inhibition has shown clinical benefit in some patients with refractory DM (6–8). Complement deposition in the microvasculature has also been associated with endothelial damage and capillary loss (9). Although both pathways may influence vascular inflammation, their relationship with neutrophil activation and coagulation in DM has not been clearly established.

Neutrophil extracellular traps (NETs) consist of extracellular chromatin associated with proteins released by neutrophils and participate in inflammatory and coagulation processes (10, 11). Previous studies in DM have reported increased NET formation in blood and affected tissues, together with impaired NET degradation (12–15). However, the composition and functional activity of these NETs have not been studied in detail. In particular, it is not known whether NETs generated in the DM inflammatory environment affect coagulation or endothelial cells.

Tissue factor (TF) initiates the extrinsic coagulation pathway and can be expressed by activated neutrophils or carried by NETs. TF-associated NETs have been described in other inflammatory conditions, where they can support thrombin generation and promote endothelial activation (16, 17). Whether DM serum induces the formation of TF-associated NETs, which serum components contribute to this response, and whether the resulting NETs can affect endothelial cells remain poorly characterized.

In this exploratory study, we investigated whether serum from patients with DM induces the formation of TF-associated NETs with procoagulant activity. The contributions of extracellular DNA/NET-associated material, C5-dependent complement activation, and serum-associated TF were examined using DNase-I, eculizumab, and TF neutralization, individually and in combination. We also assessed the effects of DM serum-induced NETs on human endothelial cells and examined lesional DM skin for TF-positive NET material. The study addressed these mechanisms under *ex vivo*, *in vitro* and *in situ* conditions and was not designed to assess clinical thrombosis, thrombotic risk, or thrombotic vasculopathy.

## Materials and methods

### Subjects

This exploratory, hypothesis-generating study enrolled seven patients within the dermatomyositis spectrum who had active skin disease. Active skin disease was defined as ongoing cutaneous inflammatory manifestations attributed to dermatomyositis by the treating clinician and documented in the medical records at the time of sampling. The diagnosis of patients was based on clinical and serological findings, and the established EULAR/ACR classification criteria (18). Muscle, skin, and lung involvement were recorded, together with autoantibody results (**Table 1**). Clinical information was obtained from the medical records available at the time of sampling. The records were reviewed for documented cutaneous vasculopathic manifestations and venous or arterial thrombotic events. Standardized vasculopathy scores, nailfold capillaroscopy findings, and formal disease-activity indices were not available. Patients with active infection or other inflammatory conditions were not included in the study. Seven sex/age-matched healthy individuals served as controls. Skin biopsies were obtained from all enrolled patients. Control skin tissue, obtained from three healthy individuals, was processed and stained in parallel using the same reagents and experimental conditions as the DM skin samples. Written informed consent was obtained from all participants. The study protocol was in accordance with the Helsinki Declaration and was approved by the Ethics Review Board of the University General Hospital of Ioannina (Approval Number:166/03.02.2026).

**Table 1:** Clinical, serological, and organ involvement characteristics of patients with dermatomyositis.

Patient	Age/Sex	Disease Duration (months)	Muscle Involvement	Cutaneous Manifestations	Pulmonary Involvement	Other Organ Involvement	Autoantibody Profile
1	73/F	1 m	Proximal muscle weakness	Heliotrope; Gottron-like rash; photosensitive rash in a V-neck distribution	Mild ILD with GGO	None	Anti-Mi2a, Anti-Mi2b, Anti-Jo1
2	24/M	1 m	Proximal muscle weakness	Non-specific erythema	Early ILD with pulmonary fibrosis	Dysphagia	Anti-MDA5, Anti-Ku
3	62/F	2 m	Proximal/distal muscle weakness	Heliotrope; Gottron-like rash; photosensitive rash in a V-neck distribution	None	Mild pericardial effusion	Anti-TIF1γ
4	77/F	3 m	None	Mild, non-specific photosensitive rash	ILD (alveolitis)	None	Anti-Jo1
5	53/F	2m	Proximal muscle weakness	Heliotrope; photosensitive rash in a V-neck distribution	None	None	Anti-Ku
6	60/F	4m	None	non-specific photosensitive rash	None	None	Anti-Mi2b, Anti-Ro52
7	71/F	2 m	Proximal muscle weakness	Cutaneous rash; heliotrope; Gottron-like rash	None	None	Anti-TIF1γ

No patient had a documented definite venous or arterial thrombotic event. Standardized vasculopathy scores and nailfold capillaroscopy data were not available at the time of sampling.

ILD, interstitial lung disease; GGO, ground-glass opacities; LAD, lymphadenopathy; NA, not available.

### Serum and plasma collection

Serum (S) and plasma EDTA (PE) were isolated from peripheral blood of dermatomyositis patients (DM) and healthy individuals (HI). In detail, venous blood was collected in proper blood collection tubes (BD Vacutainer^®^ SST II Advance Tubes, Becton, Dickinson and Company, Franklin Lakes, NJ, USA) and centrifuged at 2000xg for 10min, following manufacturer’s instructions. Peripheral blood collected in K3EDTA blood collection tubes (BD Vacutainer^®^ EDTA tubes) was centrifuged for 15min at 500xg, to isolate PE. Samples were stored at -80°C until further analysis (19, 20).

### Neutrophil isolation

Neutrophils were isolated from HI (control neutrophils). Venous blood was collected in heparinized blood collection tubes (BD Vacutainer^®^ Heparin Tubes) and neutrophil isolation was achieved by performing the Histopaque double-gradient density centrifugation (11191 and 10771, Sigma-Aldrich, St Louis, MO, USA), in accordance with manufacturer’s protocol. More precisely, whole blood was layered onto the double gradient, formed by Histopaque-1119 (Sigma-Aldrich) and Histopaque-1077 (Sigma-Aldrich), and centrifuged for 35 min at 400xg at room temperature (RT). Isolated neutrophils were washed once with 1x Dulbecco’s phosphate-buffered saline (1× PBS) (Biosera, Cholet, France) for 10 min at 200xg at RT. Cell purity of the neutrophil preparations exceeded 99%, as confirmed by May Grünwald–Giemsa stained cytospins and/or flow cytometric analysis (16).

### Culture conditions of neutrophils

Control neutrophils were cultured at 37 °C with 5% CO_2_ in Roswell Park Memorial Institute (RPMI) medium (Capricorn Scientific, Ebsdorfergrund, Germany) supplemented with 2% v/v heterologous HI serum.

### Stimulation & inhibition studies in neutrophils

Control neutrophils were stimulated using 4% v/v serum from DM patients (DM serum). Stimulation studies were performed for either 60 min to study gene expression or 3h to study NETs. The control group for all *in vitro* experiments was control neutrophils that were cultured in the absence of DM serum (17). Particularly, to explore whether DM serum-induced TF expression was modulated by pharmacological JAK inhibition, neutrophils were pre-treated with tofacitinib (10nM; 30 min, 37 °C; Cayman Chemical, Ann Arbor, MI, USA) (21). To examine the contribution of complement component C5, DM serum was treated with eculizumab (100μg/mL; 30 min, 37 °C; Selleckchem, Houston, TX, USA), prior to incubation with control neutrophils (22). Similarly, to assess the role of pre-existing extracellular DNA/NETs in serum, DM serum was pre-incubated with DNase-I (1 U/mL; 30 min, 37 °C; Thermo Fisher Scientific, Waltham, MA, USA) (16). Lastly, to inhibit TF signaling, DM serum was pre-treated with an IgG1 goat anti–human TF polyclonal antibody (10 μg/mL; 30 min, 37 °C; Sekisui Diagnostics, Burlington, MA, USA), having a neutralizing effect (16). Moreover, control neutrophils were stimulated with 4% v/v serum from patients with rheumatoid arthritis-associated interstitial lung disease (RA-ILD) (20), which served as an internal control for the thrombin-antithrombin (TAT) complex ELISA performed in isolated NETs. The concentrations and time points used to examine neutrophils were optimized before the experiments. All substances used in the study were endotoxin free, as determined by a Limulus amebocyte assay (Sigma-Aldrich).

### NET structure generation and isolation

Control neutrophils (2 x 10^6^) were seeded in a 6-well cell culture plate (SPL Life Sciences, Kyonggi-do, Republic of Korea) and maintained in RPMI medium (Capricorn Scientific) supplemented with heterologous 2% v/v HI serum. Neutrophils were subsequently stimulated with 4% v/v DM serum in the presence or absence of specific inhibitors. Following 3-hour stimulation (37 °C, 5% CO_2_), the culture medium was removed, and the cells were washed twice with pre-warmed RPMI. Fresh pre-warmed RPMI was then added and the culture plate was agitated for 3-5min to facilitate the collection of NET structures. The culture supernatant was collected and centrifuged at 20x g for 5 min at RT to remove intact cells and cellular debris. The resulting supernatant, containing the released NET structures, was collected and stored under appropriate conditions (-20 °C) until further analysis. Control neutrophils cultured under identical conditions, but without stimulation with DM serum, served as control. Where indicated, isolated NET structures were subsequently incubated with DNase-I (1 U/mL; 30 min, 37 °C; Thermo Fisher Scientific), to enzymatically degrade the DNA scaffold. Similarly, to neutralize NET-associated TF activity, NET structures were treated with a polyclonal goat IgG1 antibody against human TF (10 μg/mL; 30 min, 37 °C; Sekisui Diagnostics) (16).

### Culture conditions of human aortic endothelial cells

HAoECs were obtained from PromoCell (C-12271, PromoCell, Heidelberg, Germany) and cultured in Endothelial Cell Growth Medium MV2 (C-22020, PromoCell). Cells from passages 3 to 6 were used in experiments.

### Stimulation & inhibition studies in HAoECs

To explore the interaction between neutrophils and HAoECs, endothelial cells were exposed to *in vitro*-generated NET structures (0.5 μg DNA/mL) for 6h (16). Afterwards, cell culture supernatant was collected, centrifuged and analyzed. The concentrations and time points used to examine the interplay between neutrophils and HAoECs were optimized before the experiments. All substances used in the study were endotoxin free, as determined by a Limulus amebocyte assay (Sigma-Aldrich).

### Statistical analysis

Statistical analyses were performed using GraphPad Prism software version 8.4.2 (GraphPad Software, Inc., San Diego, CA, USA). Comparisons between HI and DM patients were performed with the use of a 2-sample independent t test (Student’s t test, 2 tailed) and means are accompanied by their 95% CIs. The nonparametric Wilcoxon test for paired samples was used to compare 2-groups (mean ± SD). For comparisons among more groups, the nonparametric Friedman’s test was used (mean ± SD). Individual observations are displayed in the quantitative panels. **Figures 1**, **2** and **Supplementary Figure S1** show means with 95% confidence intervals, whereas **Figures 3**, **4**, **5**, **6** and **Supplementary Figure S2** show means with standard deviations. These graphical summaries are descriptive. Statistical significance was assessed using the tests specified in the corresponding figure legends. The significance threshold was set at P < 0.05. Given the small sample size, the analyses were considered exploratory, and no subgroup analyses were performed. Detailed information for all methods is provided in **Supplementary Data**.

**Figure 1:**
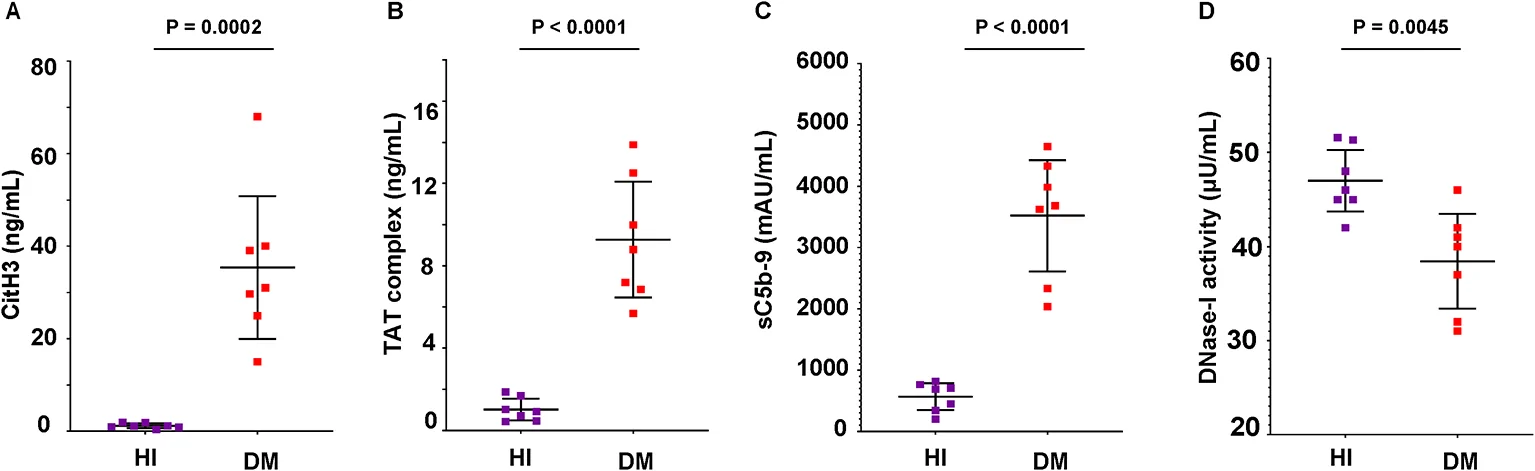
Increased levels of NET-, coagulation- and complement- related markers and reduced DNase-I activity are detected in the inflammatory milieu of patients with dermatomyositis. Concentration of **(A)** citrullinated histone H3 (CitH3), **(B)** thrombin-antithrombin (TAT) complexes, **(C)** soluble terminal complement complex (sC5b-9) and **(D)** DNase-I activity in serum/plasma samples from healthy individuals **(HI)** and dermatomyositis (DM) patients (n = 7 subjects per group). For **(A–D)**, Student’s t test (2 tailed) was used. Data are shown as means accompanied by their 95% CIs. Statistically significant: P <0.05.

**Figure 2:**
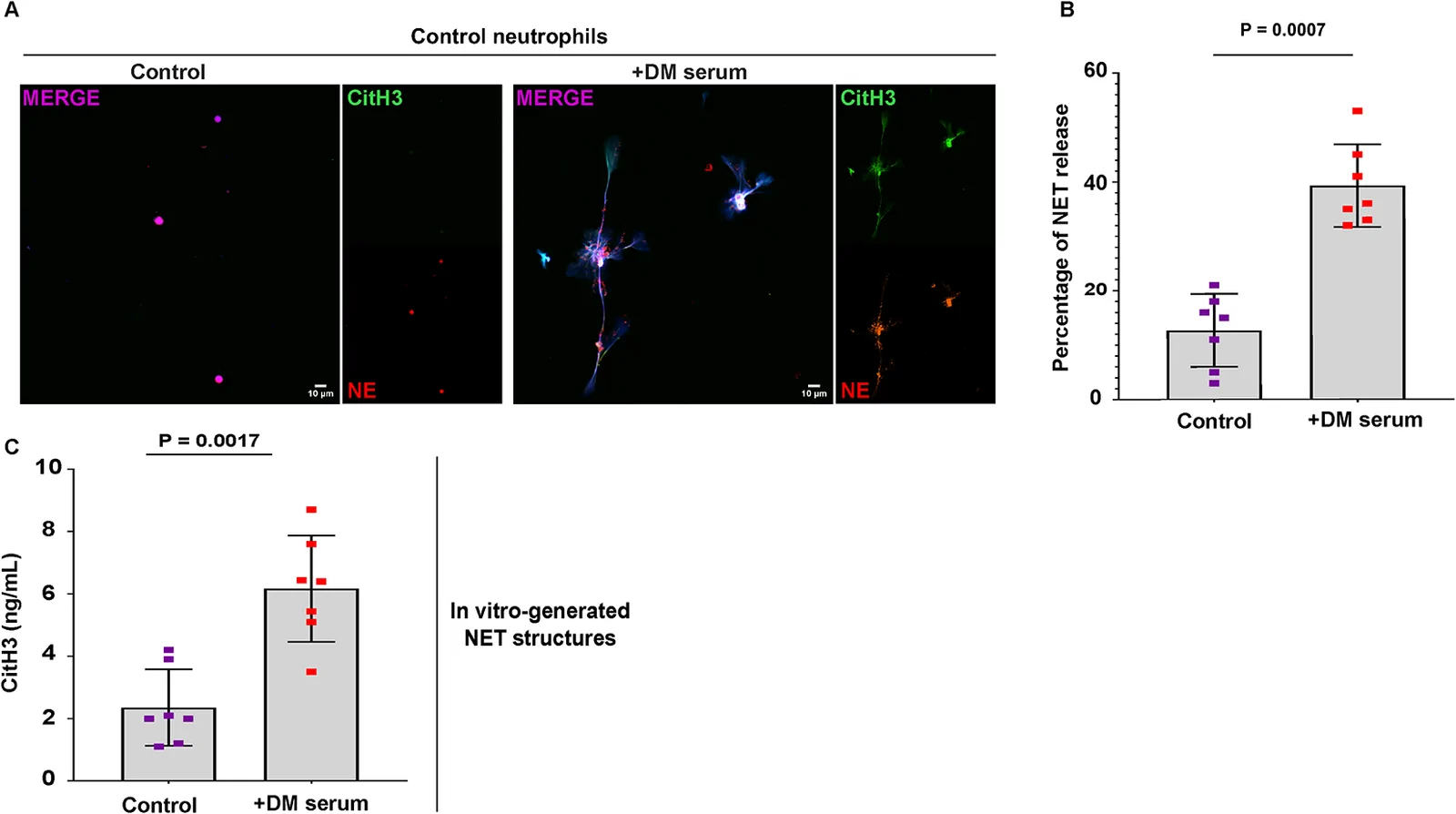
Serum from dermatomyositis patients induces the formation of NETs in control neutrophils. **(A)** Representative fluorescence confocal microscopy images showing citrullinated histone H3 (CitH3) and neutrophil elastase (NE) staining in control neutrophils or control neutrophils exposed to serum from dermatomyositis (DM) patients (*shown as* DM serum) (blue: DAPI; green: CitH3; red: NE, original magnification: 400×; optical zoom: 1.6×; scale bar: 10 μm). A representative example of 7 individual experiments is shown. **(B)** Quantification of NET releasing neutrophils, determined by examining 100 cells in a double-blind experimental procedure (n=7 independent experiments). **(C)** CitH3 levels in *in vitro* NETs isolated from either control neutrophils or control neutrophils exposed to DM serum (n=7 independent experiments). For **(B, C)**, Wilcoxon’s test for paired samples was used. Data are shown as mean ± SD. Statistically significant: P <0.05. All conditions were compared to control.

**Figure 3:**
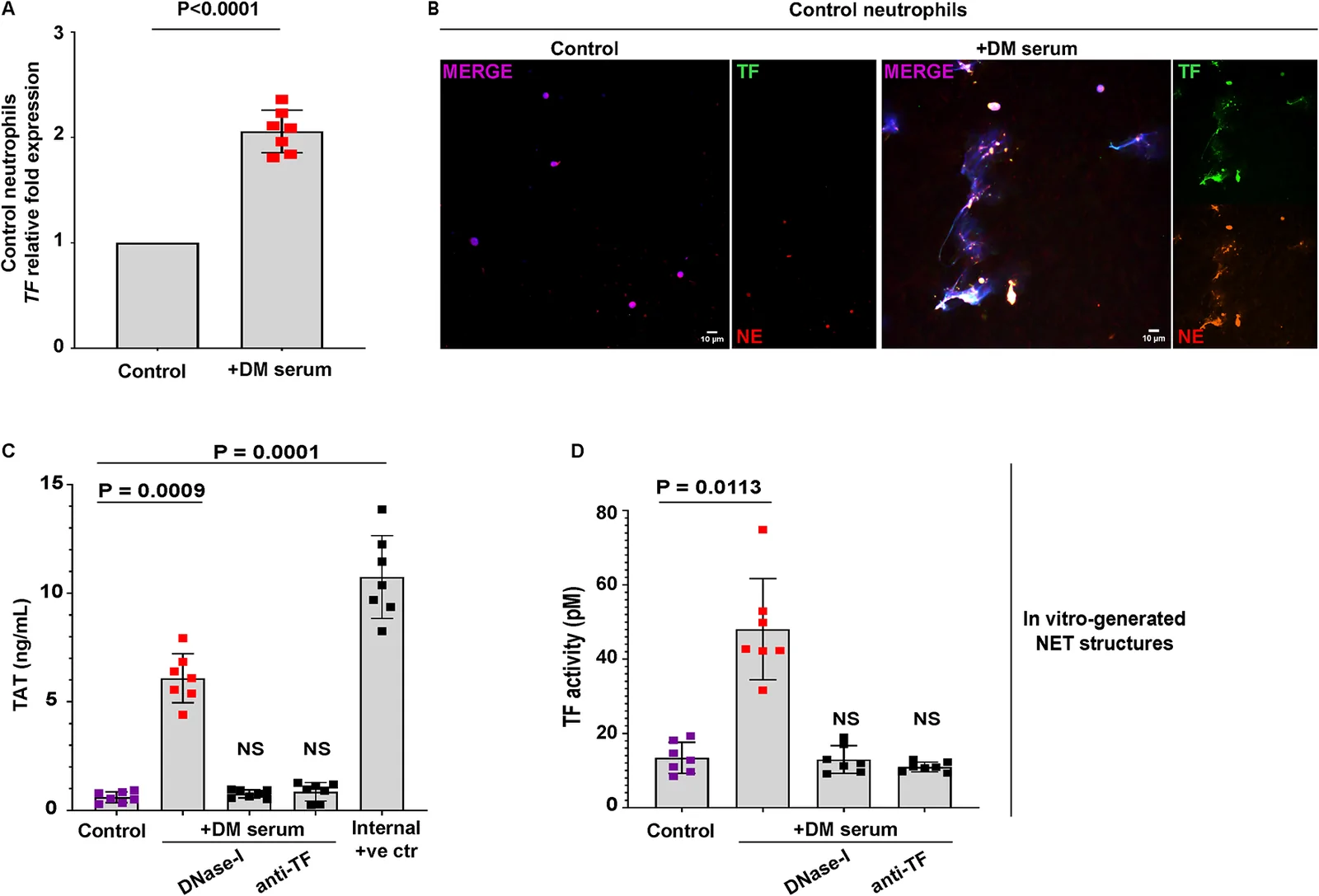
Serum from dermatomyositis patients triggers the mRNA expression of tissue factor in control neutrophils as well as the release of tissue factor-associated NETs by control neutrophils. **(A)** Tissue factor (TF) expression in control neutrophils or control neutrophils exposed to serum from dermatomyositis (DM) patients (*shown as* DM serum), as assessed by RT-qPCR (n=7 independent experiments). Wilcoxon’s test for paired samples was used. **(B)** Representative fluorescence confocal microscopy images showing TF and neutrophil elastase (NE) staining in control neutrophils or control neutrophils exposed to DM serum (blue: DAPI; green: TF; red: NE, original magnification: 400×; optical zoom: 1.6×; scale bar: 10 μm). A representative example of 7 individual experiments is shown. **(C)** TAT complex levels and **(D)** TF activity were measured in NET structures isolated from either control neutrophils or control neutrophils stimulated with DM serum. Where indicated, isolated NET structures were subsequently treated with DNase-I or a neutralizing anti-TF antibody (n=7 independent experiments). Friedman’s test was used. In **(C)**, NET structures isolated from control neutrophils, incubated with serum from patients with rheumatoid arthritis-associated interstitial lung disease (RA-ILD), served as an internal positive control (shown as Internal +ve ctr). For **(A, C, D)**, data are shown as mean ± SD. Statistically significant: P <0.05. NS, non-significant. All conditions were compared to control.

**Figure 4:**
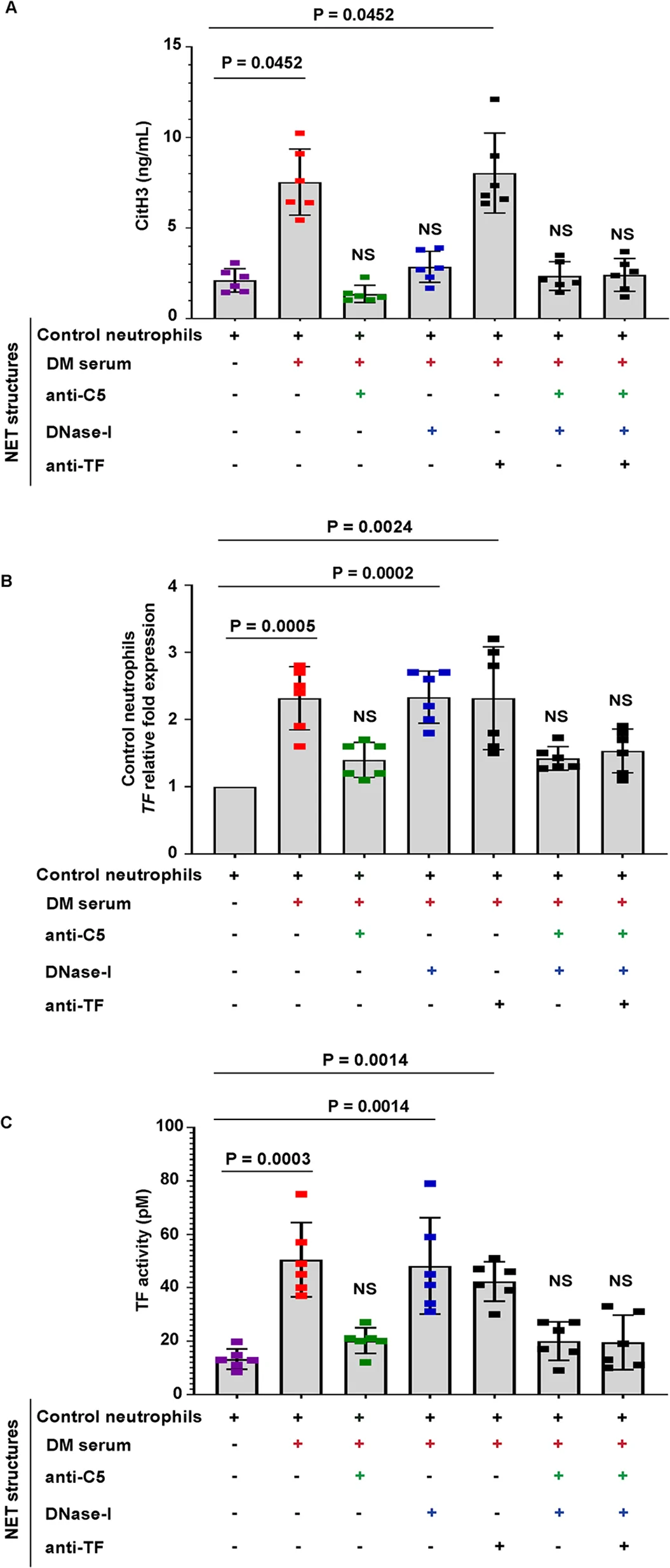
Complement component C5 contributes to NET formation and tissue factor induction in control neutrophils. **(A)** Citrullinated histone H3 (CitH3) levels were measured by ELISA in NET structures released by either control neutrophils or control neutrophils exposed to serum from patients with dermatomyositis (DM) (*shown as* DM serum). Where indicated, DM serum was pre-incubated with an anti-C5 antibody (eculizumab), DNase-I, a neutralizing anti-tissue factor (TF) antibody (*shown as* anti-TF) individually, or combinations of these agents before exposure to control neutrophils. **(B)** TF mRNA expression was assessed in control neutrophils by RT-qPCR, and **(C)** TF activity was measured by ELISA in isolated NET structures under the experimental conditions described above. For **(A–C)**, Friedman’s test was used (n=6 independent experiments). Data are shown as mean ± SD. Statistically significant: P <0.05. NS, non-significant. All conditions were compared to control.

**Figure 5:**
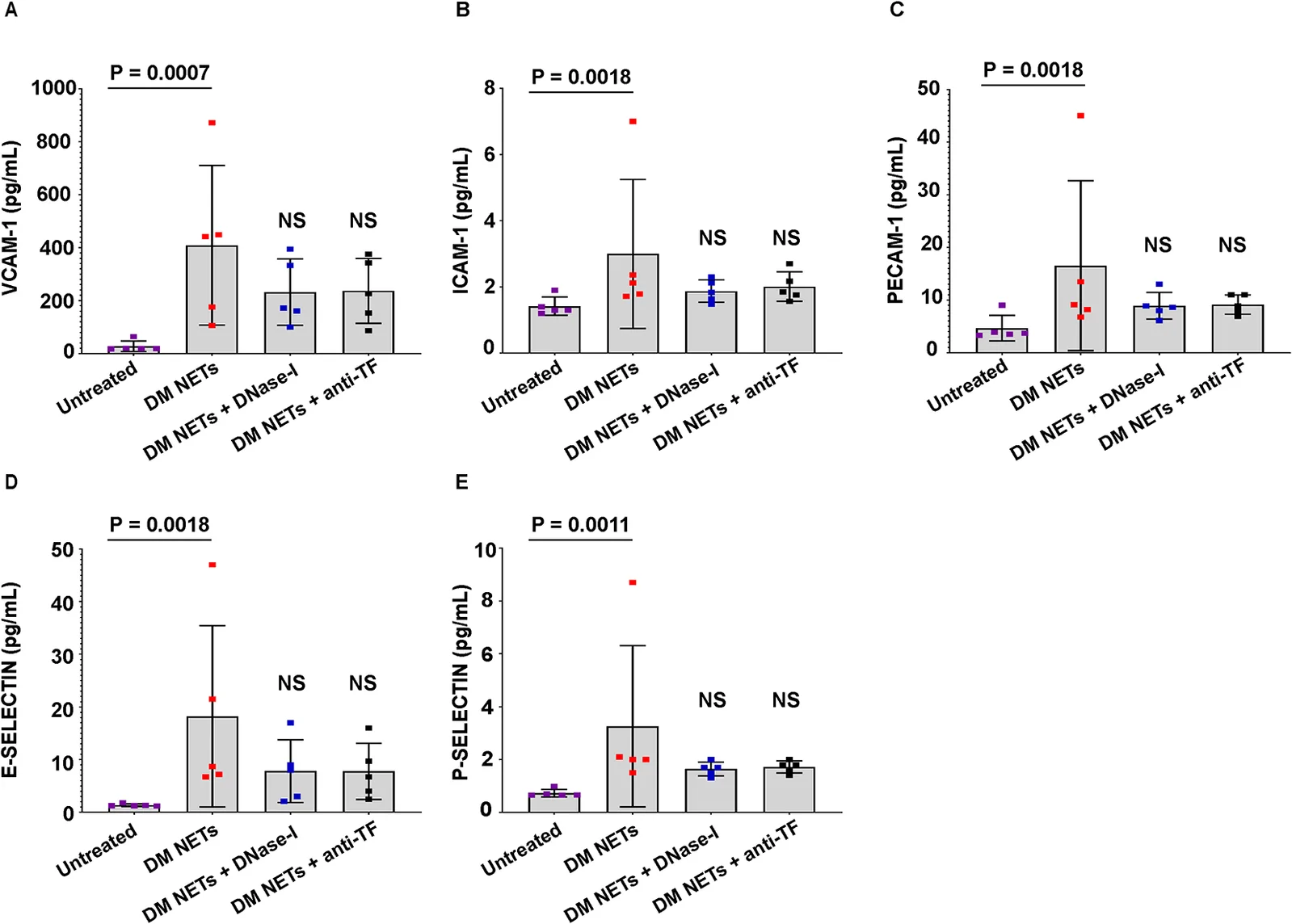
NETs induced by dermatomyositis serum promote endothelial activation *in vitro*. Human aortic endothelial cells (HAoECs) were exposed to NET structures (*shown as* DM NETs), which were secreted by control neutrophils upon stimulation with serum from dermatomyositis (DM) patients. Where indicated, isolated DM NETs were subsequently treated with DNase-I or a neutralizing anti-tissue factor (TF) antibody (*shown as* anti-TF). The levels of adhesion molecules i.e., **(A)** VCAM-1, **(B)** ICAM-1, **(C)** PECAM-1, **(D)** E-SELECTIN and **(E)** P-SELECTIN were quantified in cell supernatants by a bead-based immunoassay (n=5 independent experiments). Friedman’s test was used. Data are shown as mean ± SD. Statistically significant: P <0.05. NS, non-significant. All conditions were compared to untreated.

**Figure 6:**
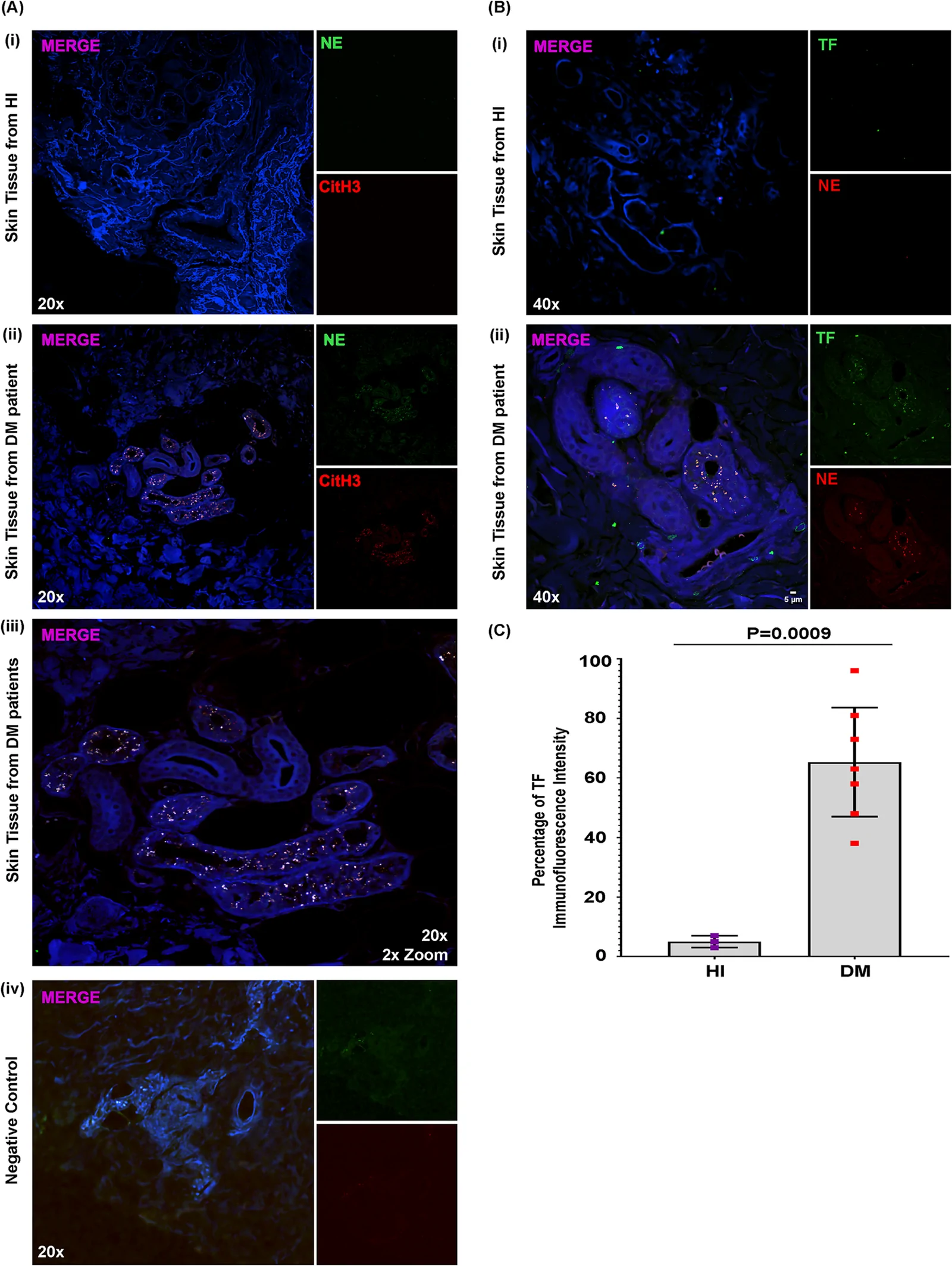
TF-positive NET fragments are present in dermatomyositis skin lesions. **(A)** Fluorescence confocal microscopy images showing neutrophil elastase (NE) and citrullinated histone H3 (CitH3) staining, representing NET remnants in skin tissue from (i) healthy individuals (HI) and (ii) patient with dermatomyositis (DM) (blue: DAPI; red: CitH3; green: NE; original magnification 200×). The tissue of DM patient is also presented with optical zoom 2x (iii). A negative control is also presented (iv). **(B)** Fluorescence confocal microscopy images showing tissue factor (TF) and NE staining, representing NET remnants enriched with TF in skin tissue from (i) HI and (ii) patient with DM (blue: DAPI; green: TF; red: NE; original magnification 400×; optical zoom 1.8x; scale bar: 5 μm). **(C)** Percentage of TF fluorescence intensity within NE-positive region. Student’s t test (2 tailed) was used. Data are shown as means accompanied by their 95% CIs. Statistically significant: P <0.05. For tissue specimens from HI: A representative example of 3 independent experiments is shown. For tissue specimens from DM: A representative example of 7 independent experiments is shown.

## Results

### Serum from dermatomyositis patients exhibits a dysregulated inflammatory profile

To characterize the circulating inflammatory milieu in DM, we performed a multiparametric protein assay in serum samples from patients with DM and healthy individuals (HI). Serum from DM patients showed significantly elevated levels of IL-6, IL-8, TNF-α, and MCP-1 compared to serum from HI (**Supplementary Figure 1**). Specific ELISA analyses also showed increased circulating levels of CitH3, thrombin–antithrombin (TAT) complexes, and soluble C5b-9 (sC5b-9) in patients with DM (**Figures 1A–C**). In contrast, DNase-I activity was significantly lower in DM serum than in serum from HI (**Figure 1D**). Together, these findings show an altered circulating inflammatory profile in DM, accompanied by increased markers of NET formation, coagulation activation, and terminal complement activation, as well as reduced DNase-I activity. In this study, increased TAT levels were considered evidence of thrombin generation and coagulation activation; clinical thrombotic outcomes and thrombotic risk were not assessed.

### Serum from dermatomyositis patients promotes NET formation in control neutrophils

Based on the presence of a dysregulated circulating inflammatory milieu in DM, we hypothesized that soluble mediators, present in DM serum, might directly modulate neutrophil function. To test this hypothesis, neutrophils isolated from healthy individuals (control neutrophils) were exposed to serum samples obtained from patients with DM. We found DM patient serum induced NET formation in control neutrophils, as demonstrated by immunofluorescence microscopy (**Figures 2A, B**) and further confirmed by elevated CitH3 levels in NETs, as measured by ELISA (**Figure 2C**). Consequently, these observations indicate that soluble factors circulating in the blood of patients with active DM can promote a NET-forming phenotype in otherwise healthy neutrophils, providing functional evidence that the DM-associated inflammatory environment may contribute to neutrophil activation.

### NETs, induced by serum from dermatomyositis patients, are enriched with tissue factor

DM has been associated with coagulation activation, although its clinical thrombotic significance remains uncertain. NETs have been implicated in thromboinflammatory processes in several inflammatory and autoimmune disorders (10, 11). We, therefore, examined whether DM serum could induce the expression of TF and the formation of TF-associated NETs by control neutrophils. As assessed by qPCR for TF, serum from patients with DM induced TF expression in control neutrophils (**Figure 3A**). Given that the functional properties of NETs are largely determined by their protein composition (10, 17), we next investigated whether TF was expressed on NETs released by control neutrophils, following stimulation with serum from patients with DM. Indeed, control neutrophils exposed to DM serum efficiently generated NETs coated with TF, as demonstrated by immunofluorescence microscopy (**Figure 3B**). Importantly, NET-associated TF was functionally active, as evidenced by increased TAT complex formation and TF activity, determined by ELISA-based assays in isolated NETs (**Figures 3C, D**). Conversely, dismantling NET structures with DNase-I or neutralizing TF on NETs significantly reduced both TAT levels and TF activity (**Figures 3C, D**). Thus, these findings indicate that serum from patients with DM induces a procoagulant NET phenotype in control neutrophils, with NET-associated TF contributing to thrombin generation.

### Eculizumab attenuates DM serum-induced NET formation and tissue factor induction

To investigate the mechanisms governing NET formation and TF expression - in response to serum from patients with DM, we performed inhibition experiments targeting complement component C5, TF, and pre-existing NETs. Specifically, DM serum was pre-incubated with eculizumab (anti-C5 antibody), anti-TF antibody, or DNase-I before exposure to control neutrophils. Eculizumab markedly reduced NET formation, as assessed by the CitH3 ELISA method (**Figure 4A**). Furthermore, the degradation of pre-existing NETs *via* DNase-I also led to a significant reduction in NETs (**Figure 4A**). Notably, neutralization of TF did not significantly affect NETs (**Figure 4A**), suggesting that serum-associated TF is not required for NET formation under the conditions tested. DNase-I, eculizumab, and anti-TF were also tested in all pairwise combinations and as a triple combination. Lower NET formation was observed in eculizumab-containing conditions; however, no statistical comparisons between inhibitor conditions were performed (**Figure 4A**). We next examined whether these interventions modulated TF expression and activity in control neutrophils. Inhibition of C5 significantly reduced TF mRNA expression, as assessed by qPCR (**Figure 4B**), and decreased TF activity on NETs (**Figure 4C**), as assessed by ELISA. In an exploratory experiment, tofacitinib also reduced DM-serum-induced TF mRNA expression (**Supplementary Figure 2**). In contrast, neither DNase-I treatment nor neutralization of serum-associated TF affected *de novo* neutrophil TF expression or the subsequent TF activity detected on NETs (**Figures 4B, C**). These findings support sensitivity of DM serum-induced NET formation and TF induction to C5 blockade under the conditions tested, but do not establish direct pathway activation or signaling hierarchy.

### TF-associated NETs promote endothelial activation *in vitro*

Vascular involvement is a recognized feature of DM (9, 23), although the mechanisms linking the inflammatory environment of DM to endothelial activation remain incompletely understood. Given the known interaction between NETs and endothelial cells (17), we examined whether NETs generated in response to DM serum could activate human endothelial cells. Hence, human aortic endothelial cells (HAoECs) were exposed to NETs generated *in vitro* by control neutrophils stimulated with DM serum (hereafter, DM NETs). DM NETs significantly increased the expression of VCAM-1, ICAM-1, PECAM-1, E-selectin and P-selectin, compared to untreated HAoECs, as assessed by a multiparametric protein assay (**Figures 5A–E**). DNase-I treatment on DM NETs markedly attenuated this response, supporting a contribution of the intact NET scaffold (**Figures 5A–E**). Neutralization of TF on DM NETs also reduced the expression of endothelial activation markers, supporting a contribution of NET-associated TF (**Figures 5A–E**). These findings show that DM serum-induced NETs promote endothelial activation *in vitro* but do not establish endothelial injury or dysfunction *in vivo*.

### Tissue factor–positive NET remnants are detected in dermatomyositis skin lesions

Given the central role of cutaneous lesions in dermatomyositis, we also examined skin tissue from patients with DM. In samples obtained from DM patients (**Supplementary Figure 3**), intact neutrophils were not observed. However, structures double-positive for neutrophil elastase (NE) and citH3 were detected in secretory coils of sweat glands (**Figure 6A**). These observed structures are consistent with NET remnants. Furthermore, TF was co-expressed within the NET fragments, as demonstrated by immunofluorescence, indicating the presence of TF-positive NET fragments in lesional DM skin (**Figures 6B, C**). In contrast, neither NET remnants nor TF-positive NET fragments were detected in skin biopsies from HI (**Figures 6A–C**), underscoring their potential association with the pathobiology of DM lesions.

## Discussion

In this exploratory study, patients with DM showed increased circulating levels of inflammatory mediators, CitH3, TAT complexes, and sC5b-9, together with reduced DNase-I activity. DM serum induced NET formation and TF expression in control neutrophils, and the resulting NETs carried functionally active TF. Isolated NET preparations showed increased TAT generation and TF activity, while disruption of the NET scaffold or direct neutralization of NET-associated TF reduced both readouts. DM serum-induced NETs also activated human endothelial cells *in vitro*, and TF-positive NET fragments were detected in lesional DM skin. These findings link serum-mediated neutrophil activation with coagulation and endothelial activation under the experimental conditions tested. They do not, however, establish clinical thrombosis, increased thrombotic risk, or thrombotic vasculopathy in patients with DM. Venous thromboembolic events have been reported in patients with DM, particularly during active disease (24). However, the present study was not designed to assess their incidence or their relationship with NET-associated TF.

Our observations are consistent with previous studies showing increased NET formation, impaired NET degradation, and neutrophil dysregulation in idiopathic inflammatory myopathies, including DM subsets with interstitial lung disease or anti-MDA5 antibodies (12, 13, 15, 25–27). The reduced DNase-I activity observed in our cohort raises the possibility that impaired extracellular DNA clearance contributes to the circulating NET burden. However, NET clearance was not directly measured, and both increased formation and reduced degradation may be involved. The contribution of these mechanisms may also differ among the clinically and serologically heterogeneous phenotypes of DM.

A central finding of this study is the presence of functionally active TF on DM serum-induced NETs. TF initiates the extrinsic coagulation pathway and has been implicated in NET-associated thrombin generation in several inflammatory conditions, including systemic lupus erythematosus and severe COVID-19 (17, 28–32). In our experiments, NET preparations - generated after exposure of healthy neutrophils to DM serum, increased both TAT generation and TF activity. DNase-I reduced these effects by disrupting the NET scaffold, while direct neutralization of TF on the isolated NETs reduced TAT generation and TF activity. The latter provides more specific evidence that NET-associated TF contributes to the procoagulant activity observed *in vitro*. TAT, nevertheless, remains a downstream marker of thrombin generation and coagulation activation (33) and should not be interpreted as evidence of clinically overt thrombosis.

The serum-interference experiments provide additional information about the factors involved in the neutrophil response. DNase-I treatment in DM serum reduced subsequent NET formation, suggesting that extracellular DNA or NET-associated material already present in the circulation may promote further release of NETs. Eculizumab reduced NET formation, TF mRNA expression, and NET-associated TF activity. In contrast, neutralization of TF already present in DM serum did not affect NET formation or *de novo* neutrophil TF expression, indicating that circulating TF was not a major driver of these responses under the conditions tested. This differs from the direct neutralization of TF on isolated NETs, which reduced their procoagulant activity.

DNase-I, eculizumab, and anti-TF were also examined individually, in all pairwise combinations, and as a triple combination. Lower NET formation was observed in eculizumab-containing conditions, although no statistical comparisons between inhibitor conditions were performed. Complement-dependent induction of neutrophil TF through C5a–C5aR signaling has previously been described in an antiphospholipid antibody model (34). This result does not establish a direct signaling sequence between complement activation, extracellular DNA, and TF, nor does it identify complement as the sole serum factor responsible for neutrophil activation. Furthermore, serum sC5b-9 was measured as a marker of terminal complement activation, whereas C5a was not measured. Although eculizumab prevents further cleavage of C5, the observed effects cannot be attributed specifically to C5a, and complement target engagement was not directly confirmed by measurement of residual complement activity after treatment.

The endothelial experiments extend the functional relevance of the NET findings. Previous studies have shown that NETs can induce endothelial adhesion molecules and TF expression in human endothelial cells (35). DM serum-induced NETs increased the expression of ICAM-1, VCAM-1, E-selectin, P-selectin, and PECAM-1 in HAoECs. This response was attenuated by DNase-I and by neutralization of NET-associated TF, supporting contributions from both the intact NET scaffold and TF. These experiments demonstrate endothelial activation *in vitro* but do not establish endothelial injury, barrier dysfunction, or microvascular damage *in vivo*. Moreover, HAoECs provide a proof-of-concept endothelial model but do not reproduce the specific properties of the cutaneous microvascular endothelium involved in DM.

The direction of the relationship between NET formation and vascular injury also remains unresolved. TF-associated NETs may amplify an established inflammatory vascular process, whereas endothelial injury and persistent inflammation may themselves promote neutrophil activation and NET release (10, 11, 23). These mechanisms are not mutually exclusive and may operate as a reciprocal process. Establishing whether TF-associated NETs initiate or amplify microvascular injury will require DM-relevant *in vivo* models in which NET formation or neutrophil TF can be selectively disrupted.

The skin findings provide further evidence that TF-associated NET material is present at sites of cutaneous disease. Semi-quantitative analysis showed greater TF-positive NET-associated staining in lesional DM skin than in healthy skin. Vasculopathy and cutaneous ulceration are recognized manifestations of DM (23, 36), making the localization of these structures potentially relevant. Nevertheless, the tissue analysis does not determine whether the NET fragments were formed locally or deposited from the circulation. It also does not demonstrate local thrombin generation, endothelial injury, or microvascular occlusion. Their functional significance within DM skin therefore remains to be established.

In an exploratory experiment, tofacitinib reduced DM-serum-induced TF mRNA expression. This finding is consistent with pharmacological sensitivity to JAK inhibition, but STAT phosphorylation and other measures of JAK/STAT target engagement were not assessed. It should therefore not be interpreted as direct evidence of JAK/STAT pathway activation or of mechanistic crosstalk between JAK/STAT and complement signaling. This distinction is important given the established type I interferon signature in DM and the emerging evidence of JAK1-associated inflammatory signaling in skin and muscle (37–39).

The translational implications of these findings are potentially important but should be framed conservatively. JAK inhibitors have shown clinical efficacy in subsets of patients with refractory DM (8, 40), whereas complement-targeted approaches remain clinically preliminary in this setting (41). The present *in vitro* findings provide a rationale for further investigation of these pathways but do not establish that modulation of TF-associated NETs contributes to clinical treatment response. TF-associated NETs should, therefore, be considered candidate mechanistic biomarkers and potential therapeutic targets rather than validated clinical biomarkers or established mediators of treatment response.

Several limitations should be considered. The cohort was small and clinically heterogeneous, precluding subgroup analyses and broad generalization across the DM spectrum. No inflammatory disease control group, non-lesional DM skin, or alternative inflammatory dermatosis was examined. Therefore, the study does not address disease specificity. The study was cross-sectional, and stimulation of healthy donor neutrophils with DM serum does not reproduce the full complexity of neutrophil activation in patients. Standardized disease-activity and vasculopathy scores, systematic nailfold capillaroscopy findings, and prospective assessment of thrombotic outcomes were not available. Correlations between NET/TF measurements and disease activity or clinical vasculopathy could therefore not be examined. None of the seven patients had a documented definite thrombotic event, and the increased TAT levels indicate coagulation activation rather than clinical thrombosis or increased thrombotic risk. In addition, C5a, STAT phosphorylation, and post-treatment complement activity were not measured. Finally, the endothelial and tissue findings do not provide evidence of vascular injury or causality *in vivo* and require confirmation in disease-relevant endothelial and animal models.

In conclusion, DM serum induced the formation of TF-associated NETs, and the isolated NET preparations increased TF activity and TAT generation *in vitro*. DM serum-induced NETs also promoted endothelial activation, and this response was attenuated by DNase-I treatment or TF neutralization. TF-positive NET fragments were also found in lesional skin. The potential relationship between NET-associated TF and disease activity, clinical vasculopathy, or thrombotic risk remains to be further investigated in larger, prospective studies.

## Data Availability

The raw data supporting the conclusions of this article will be made available by the authors, without undue reservation.
