# Supplementary material for: Tissue factor-associated neutrophil extracellular traps in dermatomyositis: procoagulant activity and endothelial activation in vitro
Source: Front Immunol. 2026 Oct 30;17:1926259. doi: 10.3389/fimmu.2026.1926259 (PMC13759836; doi:10.3389/fimmu.2026.1926259)
Supplement: Supplementary file 1 [file Supplementaryfile1.docx]

**Supplementary Data**

**Supplementary Materials & Methods**

**Immunofluorescence:** *Neutrophils.* Isolated peripheral blood neutrophils were seeded onto poly-L-lysine-coated coverslips (Biocoat, NY, USA) in a 24-well cell culture plate (SPL Life Sciences, Kyonggi-do, Republic of Korea). After 3 hours cells were fixed with 10% formaldehyde solution (Biognost, Zagreb, Croatia) for 30 min at 4 °C. Subsequently, fixed neutrophils were blocked with 6% v/v normal goat serum (Thermo Fisher Scientific, Waltham, MA, USA) in 1x PBS (blocking solution), cells were incubated (1hr, RT) with primary antibody solution, consisting of an anti-human neutrophil elastase (NE) monoclonal antibody (1:250, Abcam, Cambridge, UK) and an anti-human citrullinated histone H3 (CitH3) polyclonal antibody (1:200, Abcam) or an anti-human TF monoclonal antibody (1:200 dilution, BioMedica Diagnostics, Windsor, Canada) in blocking solution. Next, a polyclonal anti-mouse IgG AlexaFluor488 antibody (Invitrogen, Waltham, MA, USA) and a polyclonal anti-rabbit IgG AlexaFluor594 antibody (Invitrogen) were used as secondary antibodies and a 30min incubation (RT, dark) was performed. Finally, neutrophils were stained with DAPI solution (Sigma-Aldrich, St Louis, Missouri, USA) and mounted on microscope slides (Knittel Glass, Braunschweig, Germany) using a hardening mounting medium (Thermo Fisher Scientific) [1–3]. Stained neutrophils were observed under a Nikon ECLIPSE Ti2 Inverted Microscope (Nikon, Melville, NY, USA) with a 40× oil lens (1.30NA). Images were acquired with NIS-Elements software (Nikon). Image analysis was performed using Fiji software version 2.9.0 [4]. The percentage of NET-releasing cells was determined by examining 100 cells in a double-blind experimental procedure [5]. A rabbit IgG polyclonal antibody (isotype control, Abcam) and a mouse IgG polyclonal antibody (isotype control, Abcam) were used as controls.

*Formalin-fixed paraffin embedded skin biopsy sections.* Serial cross-sections (4μm each) of skin biopsies were mounted on positively charged microscope slides. Tissue sections were deparaffinized and rehydrated. Next, antigen retrieval was performed using a 10 mM Sodium Citrate buffer (pH 6.0) and non-specific binding was achieved by blocking with 2% normal goat serum (Thermo Fisher Scientific) in 2% bovine serum albumin (BSA) (Capricorn Scientific, Ebsdorfergrund, Germany) in 1x PBS (2%BSA-PBS). Following blocking, samples were incubated with primary antibody solution, consisting of anti-human NE monoclonal antibody (1:250, Abcam) and an anti-human CitH3 polyclonal antibody (1:200, Abcam) or an anti-human TF monoclonal antibody (1:200 dilution) in 2% BSA-PBS. After an overnight incubation at 4^o^C, tissue specimens were incubated for 45min with a polyclonal anti-mouse IgG AlexaFluor488 antibody (Invitrogen) and a polyclonal anti-rabbit IgG AlexaFluor594 antibody (Invitrogen) in 2% BSA-PBS. Subsequently, nuclear labeling with DAPI solution (Sigma-Aldrich, St Louis, Missouri, USA) was performed and sections were dehydrated before mounting samples with a hardening mounting medium (Thermo Fisher Scientific) and appropriate glass coverslips [6]. Stained tissue sections were observed under a Nikon ECLIPSE Ti2 Inverted Microscope (Nikon) with a 20× (0.70NA) or 40× lens (1.30NA). Images were acquired with NIS-Elements software (Nikon). Image analysis and the semi-quantitative assessment of TF fluorescence intensity, co-localized with NE, was performed using Fiji software version 2.9.0 [4], and the analyzing tool Coloc 2. NE-positive regions of interest (ROIs) were defined in the NE channel by applying an intensity threshold to separate the specific NE signal from the background. ROIs exceeding the threshold were defined as NE-positive, and the TF signal was semi-quantified within these ROIs. To dissect the co-localisation of TF-NE, thresholding was applied to both channels; TF and NE, subsequently, and the thresholded Manders coefficient (tM1) was calculated. This coefficient expresses the percentage of the total TF fluorescence signal co-localized with NE. A rabbit IgG polyclonal antibody (isotype control, Abcam) and a mouse IgG polyclonal antibody (isotype control, Abcam) were used as controls.

**Hematoxylin-Eosin staining:** To observe tissue morphology in sections of skin biopsies hematoxylin-eosin (H&E) staining was performed on tissue sections, mounted on positively charged microscope slides. In brief, sections were deparaffinized, rehydrated and stained with hematoxylin. Hematoxylin excess was rinsed with water and eosin staining followed. Samples were rinsed thoroughly with water and dehydrated. Tissue specimens were observed under an OLYMPUS IX73 Inverted Microscope (OLYMPUS Corporation, Tokyo, Japan) with a 4x air lens (0.10NA). Images were acquired using OLYMPUS cellSens Entry software version 1.14 (OLYMPUS Corporation) and processed with Fiji software version 2.9.0 [4].

**Human soluble terminal complement complex (sC5b-9) ELISA:** The levels of sC5b-9 were quantified in PE samples obtained from HI and DM patients, by utilizing a commercially available ELISA kit (Hycult Biotech, Uden, The Netherlands). The assay was performed in accordance with manufacturer’s instructions [7].

**DNase-I activity ELISA:** DNase-I activity, reflecting the enzymatic capacity for NET degradation, was quantified in serum samples obtained from HI and DM patients, using a commercially available ELISA kit (Abcam). The assay was performed according to the manufacturer’s instructions [8].

**Citrullinated histone H3 (CitH3) ELISA:** The levels of CitH3, a surrogate marker of NETs, were measured in serum samples derived from HI and DM patients. The assay also applied in *in vitro-*generated NET structures. A commercially available ELISA assay (Cayman Chemical, Ann Arbor, MI, USA) was performed in accordance with manufacturer’s instructions [1–3].

**Human thrombin-antithrombin (TAT) complex ELISA:** Plasma TAT levels were measured in samples obtained from HI and patients with DM, using a commercial-available ELISA kit (Abcam). The assay was also performed to assess the procoagulant activity of *in vitro*-generated NETs. Briefly, control plasma was stimulated with NETs, which were added at a final concentration of 20% (v/v). Plasma samples were, then, incubated for 10 min at 37°C and, immediately, placed on ice to prevent further thrombin generation. TAT complex concentrations were subsequently determined ,according to the manufacturer’s instructions [2].

**TF activity ELISA:** The levels of TF activity were assessed in *in vitro*-generated NET structures, by performing a commercially available ELISA assay (Abcam). The assay was performed based on manufacturer’s instructions [5].

**Cytokine assessment in serum samples:** Inflammatory cytokines, including IL-6, IL-8, TNF-α, and MCP-1, were quantified in serum samples obtained from HI and DM patients by applying a LEGENDplex™ Multi-Analyte Flow Assay Kit (Biolegend, San Diego, CA, USA). Samples were prepared according to manufacturer’s instructions, and data acquisition was performed in a BD Accuri C6 Plus Flow Cytometer (Becton, Dickinson and Company, Franklin Lakes, NJ, USA) [1,9].

**Adhesion molecules assessment in HAoECs supernatants:** Adhesion molecules, including ICAM-1 (CD54), VCAM-1 (CD106), E-selectin, P-selectin and PECAM-1 (CD31), were quantified in supernatants from HAoECs by applying a LEGENDplex™ Multi-Analyte Flow Assay Kit (Biolegend). Samples were prepared according to manufacturer’s instructions, and data acquisition was performed in a BD Accuri C6 Plus Flow Cytometer (Becton, Dickinson and Company) [10].

**RNA Extraction, cDNA Synthesis and Reverse Transcription qPCR:** Control neutrophils, stimulated with DM serum, were processed for RNA extraction, cDNA synthesis and reverse transcription-qPCR (RT-qPCR), as previously described [5,7,11]. Briefly, RNA isolation was achieved using TRIzol Reagent (Thermo Fisher Scientific), cDNA was synthesized with an appropriate commercial kit (Takara Bio, Shiga, Japan), and RT-qPCR was performed using a SYBR green RT-qPCR master mix (Kapa Biosystems, Wilmington, MA, USA). The normalization of gene expression was achieved with Glyceraldehyde 3-phosphate dehydrogenase (GAPDH), following the housekeeping gene normalization method. All RT-qPCR primers were designed using Beacon Designer software version 4.0. Details regarding primers and RT-qPCR conditions are provided in **Supplementary Table 1**. For RT-qPCR data analysis, the 2^−ΔΔCt^ method was applied [2,7].

**Supplementary Table 1.** Sequence of primers and RT-qPCR conditions

| **Gene** | **Primer** | **Primer Sequence** | **RT-qPCR conditions** |
| --- | --- | --- | --- |
| *TF* | FRD | 5’ AACCCGTCAATCAAGTCTACAC 3’ | 1. 52^o^C for 5 min  2. 95 ^o^C for 2 min  3. 35 cycles of:  I. 95 ^o^C for 15 sec  II. 56^o^C for 40 sec  4. 52 ^o^C for 5 min  5. Melting curve analysis |
|  | REV | 5’ TCACATCCTTCACAATCTCGTC 3’ |  |
| *GAPDH* | FRD | 5’ AGGTGGTCTCCTCTGACTTC 3’ |  |
|  | REV | 5’ CTGTTGCTGTAGCCAAATTCG 3’ |  |
| FRD: forward primer, REV: reverse primer | | | |

**Supplementary Figures**

**Supplementary Figure S1. Increased levels of inflammatory mediators in the inflammatory microenvironment of patients with dermatomyositis.** Concentrations of (**A**) IL-6, (**B**) IL-8, (**C**) MCP-1 and (**D**) TNF-a in serum samples from healthy individuals (HI) and dermatomyositis (DM) patients, as determined by a bead-based immunoassay (n = 7 subjects per group). For (**A**) - (**D**), Student’s t test (2 tailed) was used. Data are shown as means accompanied by their 95% CIs. Statistically significant: P <0.05.

**Supplementary Figure S2. Tofacitinib reduces TF expression induced by dermatomyositis serum in control neutrophils.** TF mRNA expression was assessed in control neutrophils or control neutrophils exposed to serum from patient with dermatomyositis (DM). Where indicated, DM serum was pre-incubated with tofacitinib, before exposure to control neutrophils (n = 6 subjects per group). Friedman’s test was used. Data are shown as mean ± SD. Statistically significant: P <0.05. NS: non-significant. All conditions were compared to control.

**Supplementary Figure S3. Hematoxylin-Eosin staining in a skin biopsy from patient with dermatomyositis**. Light microscopy image from a skin tissue specimen, obtained from a patient with dermatomyositis (original magnification 400x). Region marked with yellow indicates the area shown in **Figure 6** of the manuscript. A representative example of 7 independent experiments is shown.

**References**

1.	Ntinopoulou, M.; Konstantinidis, T.; Chalkidou, A.; Papagianni, E.; Skeva, A.; Panopoulou, M.; Chrysanthopoulou, A. IL-1b-Bearing NETs: Bridging Inflammation to Early Cirrhosis in Hepatitis B. International Journal of Molecular Sciences 2025, 26, 5733, doi:10.3390/ijms26125733.

2.	Venetsanopoulou, A.I.; Ntinopoulou, M.; Papagianni, E.; Koletsos, N.; Voulgari, P.V.; Chrysanthopoulou, A. Neutrophil Extracellular Traps as Immunofibrotic Mediators in RA-ILD; Pilot Evaluation of the Nintedanib Therapy. Front. Immunol. 2024, 15, doi:10.3389/fimmu.2024.1480594.

3.	Ntinopoulou, M.; Cassimos, D.; Roupakia, E.; Kolettas, E.; Panopoulou, M.; Mantadakis, E.; Konstantinidis, T.; Chrysanthopoulou, A. Ιnterleukin-17A-Enriched Neutrophil Extracellular Traps Promote Immunofibrotic Aspects of Childhood Asthma Exacerbation. Biomedicines 2023, 11, 2104, doi:10.3390/biomedicines11082104.

4.	Schindelin, J.; Arganda-Carreras, I.; Frise, E.; Kaynig, V.; Longair, M.; Pietzsch, T.; Preibisch, S.; Rueden, C.; Saalfeld, S.; Schmid, B.; et al. Fiji - an Open Source Platform for Biological Image Analysis. Nat Methods 2012, 9, 10.1038/nmeth.2019, doi:10.1038/nmeth.2019.

5.	Chrysanthopoulou, A.; Gkaliagkousi, E.; Lazaridis, A.; Arelaki, S.; Pateinakis, P.; Ntinopoulou, M.; Mitsios, A.; Antoniadou, C.; Argyriou, C.; Georgiadis, G.S.; et al. Angiotensin II Triggers Release of Neutrophil Extracellular Traps, Linking Thromboinflammation with Essential Hypertension. JCI Insight 2021, 6, e148668, doi:10.1172/jci.insight.148668.

6.	Arelaki, S.; Arampatzioglou, A.; Kambas, K.; Papagoras, C.; Miltiades, P.; Angelidou, I.; Mitsios, A.; Kotsianidis, I.; Skendros, P.; Sivridis, E.; et al. Gradient Infiltration of Neutrophil Extracellular Traps in Colon Cancer and Evidence for Their Involvement in Tumour Growth. PLoS One 2016, 11, e0154484, doi:10.1371/journal.pone.0154484.

7.	Skendros, P.; Mitsios, A.; Chrysanthopoulou, A.; Mastellos, D.C.; Metallidis, S.; Rafailidis, P.; Ntinopoulou, M.; Sertaridou, E.; Tsironidou, V.; Tsigalou, C.; et al. Complement and Tissue Factor-Enriched Neutrophil Extracellular Traps Are Key Drivers in COVID-19 Immunothrombosis. J Clin Invest 2020, 130, 6151–6157, doi:10.1172/JCI141374.

8.	Hendrix, P.; Witsch, J.; Spalart, V.; Schneider, H.; Oertel, J.; Geisel, J.; Martinod, K.; Hemmer, S. Neutrophil Extracellular Trap Biomarkers in Aneurysmal Subarachnoid Hemorrhage: Early Decline of DNase 1 Activity Associated with Delayed Cerebral Ischemia. Front. Neurol. 2024, 15, doi:10.3389/fneur.2024.1354224.

9.	Mitroulis, I.; Chrysanthopoulou, A.; Divolis, G.; Ioannidis, C.; Ntinopoulou, M.; Tasis, A.; Konstantinidis, T.; Antoniadou, C.; Soteriou, N.; Lallas, G.; et al. A Gene Expression Map of Host Immune Response in Human Brucellosis. Front Immunol 2022, 13, 951232, doi:10.3389/fimmu.2022.951232.

10.	Beckman, J.D.; Zhang, P.; Nguyen, J.; Hebbel, R.P.; Vercellotti, G.M.; Belcher, J.D. Missing the Mark(Ers): Circulating Endothelial Cells and Endothelial-Derived Extracellular Vesicles Are Elevated in Sickle Cell Disease Plasma. Front Immunol 2024, 15, 1493904, doi:10.3389/fimmu.2024.1493904.

11.	Stakos, D.A.; Kambas, K.; Konstantinidis, T.; Mitroulis, I.; Apostolidou, E.; Arelaki, S.; Tsironidou, V.; Giatromanolaki, A.; Skendros, P.; Konstantinides, S.; et al. Expression of Functional Tissue Factor by Neutrophil Extracellular Traps in Culprit Artery of Acute Myocardial Infarction. Eur Heart J 2015, 36, 1405–1414, doi:10.1093/eurheartj/ehv007.
